# Body image and self-attractiveness in a relationship during the menopausal transition: associations with digital media and perceived media influence

**DOI:** 10.3389/fpubh.2026.1829865

**Published:** 2026-06-17

**Authors:** Agnieszka Bień, Joanna Grzesik-Gąsior, Beata Górska, Beata Pięta, Krzysztof Jurek, Grażyna Bączek

**Affiliations:** 1 Chair of Obstetrics Development, Faculty of Health Sciences, Medical University of Lublin, Lublin, Poland; 2 State University of Applied Sciences in Krosno, Krosno, Poland; 3 Cardinal Stefan Wyszyński Regional Specialist Hospital, Independent Public Healthcare Centre, Lublin, Poland; 4 Department of Practical Midwifery Education, Poznan University of Medical Sciences, Poznań, Poland; 5 Institute of Sociological Sciences, Faculty of Social and Technical Sciences, John Paul II Catholic University of Lublin, Lublin, Poland; 6 Department of Obstetrics and Gynecology Didactics, Medical University of Warsaw, Warsaw, Poland

**Keywords:** body image, digital media, menopausal transition, middle-aged women, perimenopause, self-attractiveness, social media

## Abstract

**Introduction:**

This cross-sectional study examined the associations of internet and social media use and perceived media influence with body image and self-perceived attractiveness in a partner relationship among women in the menopausal transition, while accounting for the use of aesthetic medicine procedures and selected sociodemographic factors.

**Methods:**

The study included 603 women in the menopausal transition. Body image was assessed with the Body Esteem Scale (BES), and self-perceived attractiveness in a partner relationship was assessed with the Scale of Assessment of Self-Attractiveness in a Relationship (SAAR) in partnered women (*n* = 474). Linear regression models tested these associations after adjustment for selected sociodemographic variables, BMI, and other covariates.

**Results:**

Perceived media influence on body perception was more consistently associated with BES dimensions than internet and social media use. It was negatively associated with Weight Concern (β = 0.098, *p* = 0.012) and Physical Condition (β = 0.103, *p* = 0.010), whereas internet and social media use was positively associated only with Sexual Attractiveness (β = 0.092, *p* = 0.026). BMI, socioeconomic conditions, use of aesthetic medicine procedures, and age were also associated with BES outcomes. For SAAR, perceived media influence was positively associated with Body Acceptance (β = = 0.121, *p* = 0.010) and Appearance Evaluation (β = 0.114, *p* =0.015), whereas internet and social media use was positively associated with Partner Acceptance (β = 0.138, *p* = 0.003).

**Discussion:**

In women in the menopausal transition, body image was associated with both media-related and non-media factors. Perceived media influence was a more consistent correlate of body image than internet and social media use, supporting the distinction between media exposure and subjective media impact in research and care focused on body image in midlife women.

## 1 Introduction

Population aging is one of the defining demographic trends of the 21st century. Increased life expectancy and declining fertility rates mean that the number of middle-aged and older women around the world is steadily growing. It is estimated that by 2030, more than one billion women will be experiencing the menopausal transition or being postmenopausal, making menopause a significant public health challenge with implications for women's physical, mental, and social well being (1). The menopausal transition is associated with numerous hormonal and physiological changes that can affect body composition, fat distribution, skin elasticity, and sexual functioning, thereby influencing body image and feelings of attractiveness. Body image in midlife should be understood as a multidimensional construct linked to broader aspects of psychological and relational functioning, including mood, sexual functioning, and relationship satisfaction. The severity of menopausal symptoms is associated with a more negative perception of the body, and body image may partially mediate the relationship between menopausal symptoms and sexual functioning (2). These results suggest that analysis limited solely to general indicators of body image may not fully reflect the psychosocial significance of the changes occurring during the menopausal transition.

Body image is a multidimensional construct encompassing perceptual, cognitive, affective, and behavioral components of how individuals experience and evaluate their bodies. General indicators of body image typically refer to global or single-index measures that summarize overall body-related evaluation; however, such indicators may obscure domain-specific patterns. Weight- and fitness-related aspects, functional evaluations, and sexual attractiveness may be shaped by partly distinct determinants, particularly during the menopausal transition. For this reason, we assessed body image using three BES domains: Sexual Attractiveness, Weight Concern, and Physical Condition, allowing for a more nuanced interpretation of media- and context-related correlates (2). Body image in midlife is embedded in broader sociocultural norms and values, including gendered expectations regarding appearance, age-related beauty standards, and the “successful aging” ideal that emphasizes youthfulness, slimness, and vitality. Digital media can be understood as one contemporary channel through which these broader age- and gender-related appearance norms are circulated, negotiated, and reinforced. In recent years, increasing attention has been devoted to the role of social media in shaping norms regarding appearance and the aging process. Digital culture can simultaneously promote greater visibility of diverse types of appearance and reinforce ideals related to youthfulness and practices aimed at maintaining an attractive appearance (3). However, the relationship between media use and body image is complex and may be mediated by social comparison, internalization of aesthetic norms, and sensitivity to appearance-related feedback (4).

Despite the growing activity of middle-aged women in the digital space, most research on the relationship between social media and body image focuses on adolescent girls and young adult women. As a result, knowledge about the importance of digital media for the body image of women undergoing menopausal transition remains limited. However, available data suggest that it is not only the amount of time spent on social media that may be important, but also specific forms of engagement, such as posting appearance-focused content, comparing oneself to other users, or internalizing ideals of thinness and youth (5). Digital media use among midlife women is heterogeneous, ranging from passive consumption to appearance-focused engagement and comparison-based use. This heterogeneity supports the distinction between general internet/social media use and subjective perceptions of media influence on one's body image.

An important element of the contemporary sociocultural context is also the growing availability and popularity of aesthetic medicine procedures. These procedures may serve as strategies for coping with age-related changes and as indicators of investment in appearance. On the one hand, their use may be associated with greater satisfaction with one's appearance, but on the other hand, it may reflect a greater focus on appearance and greater sensitivity to the aesthetic norms present in contemporary culture (6). At the same time, social media increasingly features content related to cosmetic procedures, presented by celebrities and influencers promoting idealized body images and selfie culture. Exposure to such messages may contribute to increased body dissatisfaction, anxiety about appearance, and interest in cosmetic procedures (7).

The literature also emphasizes the multidimensional nature of body image. Its individual components may be subject to varying degrees of sociocultural influence. Aspects related to body weight and physical fitness seem to be more susceptible to aesthetic norms and social comparison processes, while the perception of sexual attractiveness may be more strongly linked to relational and psychosexual factors (8).

Despite growing interest in body image issues in midlife, there are still significant gaps in research. Previous studies rarely combine the use of digital media, the perceived influence of media representations of mature women, and the use of aesthetic medicine procedures in a single analytical model. Relatively few studies also analyze these factors in relation to both multidimensional body image and perceived self-attractiveness in partner relationships among women undergoing menopausal transition. From a clinical and public health perspective, this is particularly important because a negative body image during this period may be associated with reduced mental well being, impaired sexual functioning, and a decline in quality of life (9).

The aim of the study was to assess the associations of internet and social media use and perceived media influence with body image and self-perceived attractiveness in a partner relationship among women in the menopausal transition, while accounting for the use of aesthetic medicine procedures and selected sociodemographic factors.

## 2 Materials and methods

### 2.1 Study design and participants

The study was carried out between January 2024 and April 2025 in primary healthcare clinics located in the Lublin Province, Poland. Participants were recruited by consecutive sampling during routine clinic visits. Eligibility was determined through a structured health, gynecological, and obstetric interview that included questions on menstrual history and menopausal symptoms. Women who received information about the study and agreed to participate provided written informed consent and completed the questionnaires individually in a private setting.

Women were eligible if they were sufficiently proficient in Polish to complete the questionnaires independently. Menopausal status was established based on medical history, and participants were classified as being in perimenopause or in early postmenopause, defined as the first 12 months after the final menstrual period (FMP). The present study did not compare early and late postmenopause. The postmenopausal subgroup was restricted to women within the first 12 months after the FMP to reduce heterogeneity of menopausal stage within a cross-sectional design. An additional inclusion criterion was the self-reported presence of naturally occurring symptoms related to the menopausal transition. Classification of menopausal status was based on interview data regarding menstrual bleeding patterns and climacteric symptoms, in line with the STRAW+10 recommendations. The inclusion criterion of self-reported menopausal-transition symptoms was applied to increase the clinical relevance of the sample, as women with such symptoms frequently seek care for menopausal-transition concerns and to support interview-based classification in a cross-sectional design. We acknowledge that body composition changes may also occur in asymptomatic women; therefore, this criterion may limit the generalizability of the findings and may introduce selection bias. Because of the cross-sectional design, classification relied exclusively on interview-based information and did not include hormonal assessment or prospective confirmation of the FMP.

Women were excluded if they declined participation or withdrew consent, were more than 12 months after the FMP, did not report symptoms of the menopausal transition, were not sufficiently fluent in Polish, or failed to complete the questionnaires. Of the 621 women approached, 18 did not meet the eligibility criteria, resulting in a final sample of 603 participants. Analyses involving the Body Esteem Scale (BES) were conducted in the full sample, whereas analyses involving the Scale of Assessment of Self-Attractiveness in a Relationship (SAAR) were restricted to women who reported being in a partner relationship (*n* = 474).

The study was conducted in accordance with the Declaration of Helsinki and Good Clinical Practice guidelines. Ethical approval was granted by the Bioethics Committee of the Medical University of Lublin (Resolution No. KE-0254/265/12/2023, 14 December 2023).

### 2.2 Data collection

Data were collected using a questionnaire-based survey. The research instruments included the Body Esteem Scale (BES), the women's version of the Scale of Assessment of Self-Attractiveness in a Relationship (SAAR), and an author-developed questionnaire used to collect sociodemographic, clinical, media-related, and behavioral data. These variables included age, place of residence, relationship status, education, employment status, self-rated socioeconomic conditions, parental status, body mass index (BMI), internet and social media use, perceived media influence on body perception, and use of aesthetic medicine procedures. Education was recorded in three ordered categories: primary/vocational, secondary, and higher. Internet and social media use was assessed with a dichotomous item: “Do you use the internet and social media (e.g., Facebook, Instagram)?” (yes/no). Perceived media influence on body perception was assessed with a separate self-report item asking participants whether media portrayals of mature women influence how they perceive their own body, with response options indicating no influence, influence present, or no opinion. Use of aesthetic medicine procedures was assessed with the following author-developed dichotomous item:

“Do you currently use aesthetic medicine procedures, such as botulinum toxin, hyaluronic acid fillers, treatments for hyperpigmentation/skin-lightening procedures, laser treatments, lifting threads, aesthetic gynecology procedures, etc.?”

Responses were coded as yes/no. The item was intended to capture current use of aesthetic medicine procedures broadly, including injectable, laser-based, skin-lightening, thread-lifting, and aesthetic gynecology procedures. It did not assess the frequency, timing, or number of procedures, nor did it distinguish between specific types or levels of invasiveness. BMI was calculated from self-reported height and weight as weight in kilograms divided by height in meters squared (kg/m^2^).

The Body Esteem Scale (BES), originally developed by Franzoi and Shields and later adapted for Polish populations by Lipowska and Lipowski, was used to assess women's evaluations of their own bodies. The instrument includes 35 items scored on a 5-point Likert scale, ranging from 1 (strongly negative feelings) to 5 (strongly positive feelings). In women, the BES covers three domains: Sexual Attractiveness, Weight Concern, and Physical Condition. Higher scores indicate a more positive body-related self-evaluation. BES subscale scores were calculated as the sum of points obtained in individual items. In the Polish validation study, internal consistency was satisfactory, with Cronbach's alpha coefficients of 0.80 for Sexual Attractiveness, 0.89 for Weight Concern, and 0.82 for Physical Condition (10, 11).

The women's version of the Scale of Assessment of Self-Attractiveness in a Relationship (SAAR), developed by Chybicka and Karasiewicz, was used to measure perceived physical and sexual attractiveness within a partner relationship. The scale consists of 16 items grouped into four domains: Body Acceptance, Appearance Evaluation, Partner Acceptance, and Sexual Satisfaction. Responses are provided on a 5-point Likert scale from 1 (strongly disagree) to 5 (strongly agree). SAAR subscale scores were calculated as the mean values of responses to the items included in a given subscale, with higher scores indicating a more positive assessment of one's own attractiveness and greater acceptance in the partner relationship. Reported internal consistency for the subscales was acceptable, with Cronbach's alpha values of 0.67, 0.74, 0.71, and 0.70, respectively (12).

### 2.3 Statistical analysis

Survey data were analyzed using IBM SPSS Statistics version 28 (IBM Corp., Armonk, NY, USA). Categorical variables are presented as counts and percentages, and continuous variables as means (M) and standard deviations (SD). Associations of internet and social media use and perceived media influence on body perception with the outcomes—body image (BES subscales) and self-perceived attractiveness in a partner relationship (SAAR subscales)—were examined using hierarchical multiple linear regression. Because the dependent variables were composite scale scores rather than single Likert items, BES sum scores and SAAR mean scores were treated as approximately continuous variables for the purposes of linear regression. In Step 1, the main media-related predictors were entered: internet and social media use and perceived media influence on body perception. In subsequent steps, covariates were entered sequentially in conceptually defined blocks, including biological factors (BMI and age), sociodemographic factors (education, socioeconomic conditions, place of residence, employment status, and having children), and behavioral factors (use of aesthetic medicine procedures). This approach enabled the assessment of incremental explained variance (ΔR^2^) and changes in effect sizes after accounting for additional covariates.

Categorical variables were entered as dummy-coded predictors with clearly defined reference categories, where 0 indicated the reference group and 1 the comparison group. Coding was as follows: place of residence (0 = rural area, 1 = urban area), education (0 = primary/vocational or secondary, 1 = higher), employment status (0 = not working, 1 = working), socioeconomic conditions (0 = unsatisfactory, 1 = satisfactory), having children (0 = no, 1 = yes), and use of aesthetic medicine procedures (0 = no, 1 = yes). Internet and social media use was coded as 0 = no and 1 = yes. For regression analyses, perceived media influence on body perception was recoded into a binary predictor, with “no influence” and “no opinion” coded as 0 and “influence present” coded as 1. BMI was coded as 0 = outside the normal range and 1 = within the normal range (18.5–24.9 kg/m^2^). Model significance was evaluated using the F statistic from analysis of variance (ANOVA). For each model, unstandardized coefficients (B), standard errors (SE), standardized coefficients (β), *t* values, *p* values, 95% confidence intervals (CI) for B, and variance inflation factors (VIF) were reported. Model fit was assessed using the multiple correlation coefficient (*R*), coefficient of determination (*R*^2^), adjusted R^2^, and ΔR^2^ across sequential steps. Statistical significance was set at α = 0.05. Multicollinearity was assessed using VIF values. The assumptions of linear regression, including linearity, normality of residuals, and homoscedasticity, were checked and no substantial violations were identified.

For transparency and reproducibility, full hierarchical model outputs for the variables retained in the sequential models are provided in the Supplementary Tables. The main manuscript tables present the final models in a concise format to enhance readability.

Questionnaires were checked for completeness at the time of administration, and participants were given the opportunity to complete missing responses. Consequently, missing data were minimal. Regression models were estimated using a complete-case approach, with listwise deletion applied separately for each model.

## 3 Results

BES subscales were computed as sum scores, with higher scores indicating a more favorable body evaluation. SAAR analyses were conducted in partnered women (*n* = 474). SAAR subscales were computed as mean scores across four items per domain, with higher scores indicating a more favorable perception in the relationship context. The mean age of the participants was 50.39 years (SD = 3.48; median = 50.5). Most women lived in urban areas, with 43.0% residing in a regional capital and 34.8% in another city. Nearly half had higher education (47.9%), 78.6% were in a stable relationship, and 85.6% were professionally active. Most participants rated their socioeconomic conditions as satisfactory (74.1%) and had children (71.8%). More than half reported no comorbidities (54.4%), and three-quarters declared no tobacco or alcohol use (75.6%). In terms of BMI, 50.9% had normal body weight. The majority reported using the internet and social media (87.7%), 34.7% declared that media influenced their perception of their own body, and 15.9% reported current use of aesthetic medicine procedures (Table 1).

**Table 1:** Sample characteristics.

Sociodemographic data		*N*	%
Age (years) *M* (Me) ± SD	*M* = 50.39, Me = 50.5, SD = 3.48		
Place of residence	Regional capital	259	43.0
	Other city	210	34.8
	Rural area	134	22.2
Education	Primary/vocational	56	9.3
	Secondary	258	42.8
	Higher	289	47.9
Relationship status	In a stable relationship	474	78.6
	Single	129	21.4
Employment status	Employed	516	85.6
	Unemployed	36	6.0
	On disability pension/retired	51	8.4
Self-rated socioeconomic conditions	Satisfactory	447	74.1
	Unsatisfactory	156	25.9
Having children	No	170	28.2
	Yes	433	71.8
Comorbidities	No	328	54.4
	Yes	275	45.6
Tobacco and alcohol use	No	456	75.6
	Yes	147	24.4
BMI	Underweight (< 18.5)	5	0.8
	Normal weight (18.5–24.9)	307	50.9
	Overweight (25.0–29.9)	190	31.5
	Obesity (≥30.0)	101	16.7
Internet and social media use	Yes	529	87.7
	No	74	12.3
Perceived media influence on body perception	No influence	261	43.3
	Influence present	209	34.7
	No opinion	133	22.1
Use of aesthetic medicine procedures	No	507	84.1
	Yes	96	15.9

Descriptive statistics showed that Sexual Attractiveness had the highest mean score among the BES subscales (*M* = 43.61, Me = 44.0, SD = 8.68), whereas the mean scores for Weight Concern and Physical Condition were 31.50 (Me = 31.0, SD = 8.21) and 29.68 (Me = 30.0, SD = 6.78), respectively. SAAR scores showed limited variation across subscales. The lowest mean score was observed for Body Acceptance (*M* = 2.93, Me = 3.0, SD = 0.35), while the highest was found for Appearance Evaluation (*M* = 3.11, Me = 3.0, SD = 0.38). The mean scores for Partner Acceptance and Sexual Satisfaction were 3.06 (Me = 3.0, SD = 0.40) and 3.03 (Me = 3.0, SD = 0.42), respectively.

Table 2 presents the final linear regression models for the three Body Esteem Scale (BES) subscales: Sexual Attractiveness, Weight Concern, and Physical Condition. Full hierarchical model outputs for the variables retained in the sequential models are provided in the Supplementary Tables. All final BES models were statistically significant and explained between 12.4 and 18.4% of the variance in the outcomes. Overall, the pattern was domain-specific: perceived media influence was associated with weight- and fitness-related body esteem domains, whereas general internet and social media use was associated only with Sexual Attractiveness. In the final model for Sexual Attractiveness, internet and social media use was positively associated with the outcome (β = 0.092, *p* = 0.026), whereas perceived media influence on body perception was not significant (β = −0.032, *p* = 0.429). Across successive steps, a gradual increase in explained variance was observed. Adding BMI in Model 2 increased adjusted *R*^2^ from 3.0 to 6.2%, suggesting that BMI contributed to improved model fit for Sexual Attractiveness. Including education in Model 3 was associated with a further increase in adjusted *R*^2^ to 8.3%. Adding use of aesthetic medicine procedures in Model 4 increased adjusted *R*^2^ to 9.6%, whereas inclusion of socioeconomic conditions in Model 5 increased adjusted *R*^2^ to 10.9%. The final model, additionally adjusted for age, reached an adjusted *R*^2^ of 11.4%. The largest increment in explained variance was observed after BMI was entered, while subsequent blocks contributed progressively smaller, though still noticeable, increments to model fit. At the same time, standardized coefficients for the main predictors changed only moderately across steps, suggesting relative stability of the associations after accounting for successive covariate blocks. For Weight Concern, perceived media influence on body perception was negatively associated with the outcome (β = −0.098, *p* = 0.012), while internet and social media use was not significant (β = 0.030, *p* = 0.448). Across successive steps, a clear increase in explained variance (adjusted *R*^2^) was observed. Adding BMI in Model 2 increased adjusted *R*^2^ from 1.6 to 14.8%. Adding use of aesthetic medicine procedures in Model 3 was associated with a further increase in adjusted *R*^2^ to 16.2%. Including place of residence in Model 4 increased adjusted *R*^2^ to 16.9%, whereas adding socioeconomic conditions in Model 5 increased adjusted *R*^2^ to 17.6%. The largest increment in explained variance occurred after BMI was entered, while subsequent variables contributed progressively smaller improvements in model fit. This suggests that BMI appeared to be the main contributor to the increase in model fit for Weight Concern, whereas social and behavioral variables provided additional explanatory value. At the same time, the association between internet and social media use and Weight Concern observed in the initial model attenuated after inclusion of covariates, suggesting that this relationship may be partly accounted for by other factors included in the models. A similar pattern was observed for Physical Condition, where perceived media influence on body perception was negatively associated with the outcome (β = −0.103, *p* = 0.010), whereas internet and social media use was not significant (β = 0.010, *p* = 0.808). Across successive steps, a systematic increase in explained variance (adjusted *R*^2^) was observed. Adding BMI in Model 2 increased adjusted *R*^2^ from 1.4 to 6.8%, suggesting that BMI contributed to improved model fit for Physical Condition. Adding use of aesthetic medicine procedures in Model 3 was associated with a further increase in adjusted *R*^2^ to 9.1%. Including socioeconomic conditions in Model 4 increased adjusted *R*^2^ to 10.4%, whereas adding age in Model 5 increased adjusted *R*^2^ to 11.5%. In subsequent steps, the incremental gain became progressively smaller: adding place of residence in Model 6 increased adjusted *R*^2^ to 12.3%, and including education in the final model increased adjusted *R*^2^ to 12.8%. The largest increment in explained variance occurred after BMI was entered, while subsequent variables contributed diminishing, though still noticeable, improvements in model fit. This suggests that BMI appeared to account for the largest increase in model fit for Physical Condition, whereas sociodemographic and behavioral variables provided additional explanatory value. At the same time, relatively small changes in the regression coefficients for the main predictors across successive models suggest that the observed associations were broadly stable after accounting for covariates. In addition, BMI and use of aesthetic medicine procedures were significant in all three models, socioeconomic conditions were significant in all final BES models, and age was significant for Sexual Attractiveness and Physical Condition. Given the coding, positive BMI coefficients indicate higher outcome scores among women with BMI in the normal range compared with those outside the normal range. Positive education coefficients indicate higher outcome scores among women with higher education compared with those with primary/vocational or secondary education.

**Table 2:** Final linear regression models for the three Body Esteem Scale (BES) subscales, including media-related, sociodemographic, and behavioral variables.

Model	*B*	SE	β	*t*	*p*	95% CI LL	95% CI UL	VIF
*Sexual attractiveness F*(7, 595) = 12.015, *p* < 0.001, *R* = 0.352, *R*^2^ = 12.4%, adjusted *R*^2^ = 11.4%								
(Constant)	47.727	5.328		8.958	0.000	37.264	58.191	
Internet and social media use	2.427	1.085	0.092	2.236	0.026	0.295	4.559	1.144
Perceived media influence on body perception	−0.580	0.731	−0.032	−0.792	0.429	−2.016	0.857	1.093
BMI	2.384	0.687	0.137	3.470	**0.001**	1.035	3.733	1.064
Education	2.057	0.709	0.118	2.903	**0.004**	0.665	3.449	1.131
Use of aesthetic medicine procedures	3.005	0.956	0.127	3.143	**0.002**	1.127	4.883	1.104
Self-rated socioeconomic conditions	2.510	0.793	0.127	3.166	**0.002**	0.953	4.067	1.087
Age	−0.209	0.100	−0.083	−2.093	**0.037**	−0.406	−0.013	1.066
*Weight concern F*(6, 596) = 22.470, *p* < 0.001, *R* = 0.429, *R*^2^ = 18.4%, adjusted *R*^2^ = 17.6%								
(Constant)	25.431	1.026		24.784	0.000	23.416	27.446	
Internet and social media use	0.748	0.985	0.030	0.760	0.448	−1.187	2.683	1.136
Perceived media influence on body perception	−1.682	0.665	−0.098	−2.531	**0.012**	−2.988	−0.377	1.088
BMI	5.485	0.623	0.334	8.806	**0.000**	4.261	6.708	1.054
Use of aesthetic medicine procedures	2.792	0.864	0.125	3.233	**0.001**	1.096	4.488	1.086
Place of residence	1.894	0.757	0.096	2.501	**0.013**	0.407	3.382	1.078
Self-rated socioeconomic conditions	1.731	0.712	0.092	2.430	**0.015**	0.332	3.129	1.058
*Physical condition F*(8, 594) = 12.09, *p* < 0.001, *R* = 0.374, *R*^2^ = 14.0%, adjusted *R*^2^ = 12.8%								
(Constant)	36.400	4.139		8.795	0.000	28.271	44.528	
Internet and social media use	0.208	0.858	0.010	0.243	0.808	−1.476	1.892	1.189
Perceived media influence on body perception	−1.469	0.567	−0.103	−2.590	**0.010**	−2.584	−0.355	1.095
BMI	2.452	0.534	0.181	4.591	**0.000**	1.403	3.501	1.071
Use of aesthetic medicine procedures	2.643	0.743	0.143	3.558	**0.000**	1.184	4.102	1.110
Self-rated socioeconomic conditions	1.686	0.615	0.109	2.743	**0.006**	0.479	2.893	1.088
Age	−0.218	0.078	−0.111	−2.815	**0.005**	−0.371	−0.066	1.066
Place of residence	1.505	0.646	0.092	2.330	**0.020**	0.236	2.775	1.084
Education	1.149	0.551	0.085	2.085	**0.037**	0.067	2.231	1.138

B—unstandardized coefficient; SE—standard error; β–standardized coefficient; 95% CI-−95% confidence interval; VIF—variance inflation factor; F—analysis of variance (ANOVA) statistic; R—multiple correlation coefficient; R^2^—coefficient of determination. Values in bold indicate statistical significance at the *p* < 0.05 level.

Table 3 presents the final linear regression models for the four Scale of Assessment of Self-Attractiveness in a Relationship (SAAR) subscales: Body Acceptance, Appearance Evaluation, Partner Acceptance, and Sexual Satisfaction. Full hierarchical model outputs for the variables retained in the sequential models are provided in the Supplementary Tables. All final SAAR models were statistically significant, although they explained only a small proportion of the variance in the outcomes (*R*^2^ ranging from 2.1 to 3.9%). Overall, associations were more selective than in the BES models, suggesting that additional relational and psychosexual factors not captured in the present models may contribute to these outcomes. For Body Acceptance, perceived media influence on body perception (β = 0.121, *p* = 0.010) and use of aesthetic medicine procedures (β = 0.126, *p* = 0.007) were positively associated with the outcome, whereas internet and social media use was not significant (β = 0.011, *p* = 0.819). In the sequential analysis, explained variance increased after the inclusion of additional predictors. In Model 1, adjusted *R*^2^ was 1.9%. Adding use of aesthetic medicine procedures in Model 2 increased adjusted *R*^2^ to 3.2% (Δadjusted *R*^2^ = 1.3%). These results indicate that although perceived media influence and use of aesthetic medicine procedures were statistically significantly associated with the outcome, their combined contribution to explaining its variability remained relatively small. For Appearance Evaluation, perceived media influence on body perception (β = 0.114, *p* = 0.015) and professional activity (β = 0.105, *p* = 0.025) were positively associated with the outcome, while internet and social media use was not significant (β = −0.006, *p* = 0.906). A small increase in explained variance was observed after adding subsequent predictors. In Model 1, adjusted *R*^2^ was 1.0%. Adding employment status in Model 2 increased adjusted *R*^2^ to 1.8% (Δadjusted *R*^2^ = 0.8%). The findings suggest that perceived media influence and employment status were statistically significantly associated with the outcome; however, their combined contribution to explaining its variability was very limited. For Partner Acceptance, internet and social media use (β = 0.138, *p* = 0.003), age (β = 0.117, *p* = 0.012), and use of aesthetic medicine procedures (β = 0.116, *p* = 0.014) were positively associated with the outcome, whereas perceived media influence on body perception was not significant (β = −0.050, *p* = 0.290). A gradual, though modest, increase in explained variance was observed as additional predictors were entered. In Model 1, adjusted *R*^2^ was 1.2%. Adding age in Model 2 increased adjusted *R*^2^ to 2.0% (Δadjusted *R*^2^ = 0.8%). Including use of aesthetic medicine procedures in Model 3 was associated with a further increase in adjusted *R*^2^ to 3.1% (Δadjusted *R*^2^ = 1.1%). Overall, internet and social media use, but not perceived media influence, and selected demographic/behavioral factors were significantly associated with the outcome, yet their combined contribution to explaining its variability remained small. For Sexual Satisfaction, neither internet and social media use (β = 0.004, *p* = 0.937) nor perceived media influence on body perception (β = −0.009, *p* = 0.852) was statistically significant. In the hierarchical analysis, the initial model including only media-related variables was not significant and explained virtually no variance in the dependent variable (adjusted *R*^2^ = −0.4%). Adding having children in Model 2 significantly improved model fit and increased adjusted *R*^2^ to 1.5% (Δadjusted *R*^2^ = 1.9%). Having children was the only significant predictor and was negatively associated with Sexual Satisfaction (β = −0.146, *p* = 0.002), indicating that media-related factors did not play a meaningful role in explaining this outcome.

**Table 3:** Final linear regression models for the four Scale of Assessment of Self-Attractiveness in a Relationship (SAAR) subscales, including media-related, sociodemographic, and behavioral variables.

Model	*B*	SE	β	*t*	*p*	95% CI LL	95% CI UL	VIF
*Body acceptance F*(3, 470) = 6.230, *p* < 0.001, *R* = 0.196, *R*^2^ = 3.8%, adjusted *R*^2^ = 3.2%								
(Constant)	2.873	0.046		61.902	0.000	2.782	2.964	
Internet and social media use	0.011	0.050	0.011	0.228	0.819	−0.087	0.110	1.040
Perceived media influence on body perception	0.088	0.034	0.121	2.578	**0.010**	0.021	0.155	1.084
Use of aesthetic medicine procedures	0.118	0.043	0.126	2.717	**0.007**	0.033	0.203	1.054
*Appearance evaluation F*(3, 470) = 3.942, *p* = 0.009, *R* = 0.157, *R*^2^ = 2.5%, adjusted *R*^2^ = 1.8%								
(Constant)	2.981	0.063		47.636	0.000	2.858	3.104	
Internet and social media use	−0.007	0.056	−0.006	−0.118	0.906	−0.118	0.104	1.089
Perceived media influence on body perception	0.090	0.037	0.114	2.453	**0.015**	0.018	0.163	1.037
Employment status	0.121	0.054	0.105	2.251	**0.025**	0.015	0.227	1.052
*Partner acceptance F*(4, 469) = 4.742, *p* < 0.001, *R* = 0.197, *R*^2^ = 3.9%, adjusted *R*^2^ = 3.1%								
(Constant)	2.229	0.280		7.971	0.000	1.680	2.779	
Internet and social media use	0.172	0.058	0.138	2.975	**0.003**	0.058	0.286	1.055
Perceived media influence on body perception	−0.041	0.039	−0.050	−1.059	0.290	−0.118	0.035	1.084
Age	0.013	0.005	0.117	2.532	**0.012**	0.003	0.024	1.041
Use of aesthetic medicine procedures	0.124	0.050	0.116	2.478	**0.014**	0.026	0.223	1.073
*Sexual satisfaction F*(3, 470) = 3.388, *p* = 0.018, *R* = 0.145, *R*^2^ = 2.1%, adjusted *R*^2^ = 1.5%								
(Constant)	3.152	0.069		45.463	0.000	3.016	3.288	
Internet and social media use	0.005	0.061	0.004	0.079	0.937	−0.114	0.124	1.038
Perceived media influence on body perception	−0.008	0.041	−0.009	−0.186	0.852	−0.087	0.072	1.043
Having children	−0.150	0.047	−0.146	−3.180	**0.002**	−0.243	−0.057	1.009

B—unstandardized coefficient; SE—standard error; β–standardized coefficient; 95% CI−95% confidence interval; VIF—variance inflation factor; F—analysis of variance (ANOVA) statistic; R—multiple correlation coefficient; R^2^—coefficient of determination. Values in bold indicate statistical significance at the *p* < 0.05 level.

## 4 Discussion

This study examined associations between internet and social media use, perceived influence of media portrayals of mature women on body perception, and multidimensional body image, as well as self-perceived attractiveness in a partner relationship, among women in the menopausal transition. The results indicate that these associations were selective and varied depending on the domain analyzed. A more consistent pattern was observed for body image (BES) than for relational self-attractiveness (SAAR). The perceived influence of media representations of mature women was more consistently associated across BES domains than media use itself. BES models explained a moderate proportion of the variance, indicating that, beyond the predictors analyzed, additional psychological and health-related factors may also be relevant. The observed associations were domain-specific and effect sizes were modest, consistent with the moderate explained variance for BES and the small explained variance for SAAR outcomes.

### 4.1 Body esteem during the menopausal transition

With regard to body image, the more pronounced associations were observed for Weight Concern and Physical Condition rather than Sexual Attractiveness. This pattern can be interpreted in the light of sociocultural approaches, according to which aspects of body image related to weight, fitness, and signs of aging are particularly susceptible to aesthetic norms and social comparisons. While digital media represent an important contemporary context, body image in midlife is also shaped by broader sociocultural forces, including age-related appearance norms, gendered expectations, and ideals emphasizing youthfulness, slimness, fitness, and active aging. These broader norms may influence how women interpret bodily changes during the menopausal transition and may contribute to domain-specific vulnerabilities, particularly in relation to weight- and fitness-related body concerns (4, 8, 13, 14).

During the menopausal transition, when somatic changes become more noticeable, media messages promoting slimness, youth, and fitness may be particularly salient for body image evaluation (3, 5). At the same time, the more limited associations observed for sexual attractiveness may suggest that this dimension of body image remains more strongly embedded in a broader psychosexual and relational context. It is worth noting, however, that the digital environment can be not only a source of pressure to look a certain way, but also a space for self-presentation, social interaction, and exposure to more diverse representations of femininity and aging, which may partly explain the more complex nature of the relationships observed for sexual attractiveness (15, 16).

Body image during the menopausal transition is multifactorial and remains embedded in a broader biopsychosocial context. The study particularly highlighted the role of sociodemographic factors, indicating that the way women perceive their bodies during this period depends not only on hormonal and somatic changes, but also on social, material, and environmental resources that can strengthen or weaken well being (17).

Socioeconomic conditions were associated with all analyzed domains of body image. This suggests that women's body image during the menopausal transition is embedded not only in the experience of biological changes, but also in the context of material and psychosocial resources that may influence lifestyle, access to health-promoting activities, opportunities to engage in appearance-related practices, and a sense of agency in relation to age-related changes. Better socioeconomic conditions may support a more positive body image through greater access to health resources, opportunities for physical activity, and behaviors related to health and appearance. From a public health perspective, this is consistent with evidence indicating that the course of the menopausal transition and the severity of menopausal symptoms vary by social and economic conditions, which may also indirectly translate into mental well being and body image (1, 17).

The associations with age for selected domains of body image can be interpreted as reflecting changes related to life stage and the course of the menopausal transition. With age, changes in body composition, physical fitness, muscle mass, and strength intensify, which may affect the subjective evaluation of physical condition and functional aspects of the body (2, 18). At the same time, the association between age and the assessment of sexual attractiveness can be understood in the context of broader psychosexual conditions during this period. Studies on middle-aged women indicate that a sense of attractiveness remains an important component of sexual well being and may be particularly sensitive to physical changes, menopausal symptoms, and accompanying emotional factors (15, 16). When considering weight-related concerns, however, ways of relating to appearance norms, experiences of social comparison, and practices aimed at maintaining a desirable appearance may be more important (13, 14).

When interpreting the results, it is also worth considering the role of education and place of residence. A higher level of education may be interpreted as an indicator of cognitive and health-related resources conducive to a more adaptive perception of one's own body, greater health awareness, and more frequent engagement in behaviors that support fitness (19, 20). In this sense, education may act not only as a traditional indicator of social status, but also as a resource supporting health competencies and behaviors conducive to well being during the menopausal transition (21).

In turn, the importance of place of residence suggests that the living environment may shape body-related experiences in midlife. Differences between areas with varying degrees of urbanization may reflect unequal access to infrastructure supporting physical activity and a healthy lifestyle, as well as differences in social norms regarding appearance, fitness, and aging (22, 23). From a public health perspective, this finding emphasizes that body image should not be treated solely as an individual psychological trait, but also as a phenomenon embedded in the social and spatial environment.

Also noteworthy is the importance of aesthetic medicine procedures, which may reflect active investment in appearance in response to age- and menopause-related changes. In this context, these procedures can be understood not only as appearance-oriented practices, but also as attempts to maintain a sense of agency in the face of bodily changes associated with aging and the hormonal transition (24, 25). On the one hand, such practices may be associated with the desire to maintain a sense of attractiveness and a more positive body evaluation; on the other hand, they are embedded in a broader cultural context in which appearance remains an important element of female identity, and digital media may reinforce attention to appearance and interest in aesthetic procedures (3, 4, 7).

The associations with BMI further indicate that body image during the menopausal transition remains strongly linked to the somatic dimension, although these relationships should be interpreted in the context of co-occurring social, behavioral, and cultural factors. This is consistent with earlier observations that menopause and the physical changes that accompany it may be associated with a less favorable perception of the body, with body image simultaneously shaped by broader psychosocial and cultural conditions (2, 8, 19).

These results indicate that women's body image during the menopausal transition is shaped by overlapping biological, social, and cultural influences. In practice, this means that a broader, more integrated approach is needed to support the well being of middle-aged women, taking into account not only menopausal symptoms and lifestyle, but also social resources, living conditions, health literacy, and the cultural context related to norms of appearance and aging (1, 9).

### 4.2 Self-perceived attractiveness in a relationship during the menopausal transition

In comparison with the findings related to body image, the associations observed for self-perceived attractiveness in a relationship were more selective and less consistent. This may suggest that, during the menopausal transition, the relational dimension of perceived attractiveness is less directly linked to media-related factors and more strongly embedded in a broader psychosexual, emotional, and relational context. This interpretation is consistent with evidence showing that women's experiences of attractiveness and sexual well being in midlife are shaped not only by body appearance, but also by relationship quality, emotional support, and broader psychosocial functioning (26).

It is noteworthy that the perceived influence of media portrayals of mature women was associated with dimensions related to body and appearance evaluation. This may suggest that media representations of aging femininity primarily affect how women evaluate their own bodies and appearance, rather than directly influencing the relational dimension of attractiveness. Importantly, this finding does not necessarily indicate a purely negative role of media. Women who acknowledge the relevance of media messages may also be more engaged in appearance-related self-care practices or more likely to compare their appearance with broader cultural standards of femininity and aging (4, 27). Contemporary media environments may therefore function not only as sources of appearance pressure, but also as spaces in which more diverse representations of women's bodies and aging are encountered (28). The association between internet and social media use and the more relational dimension of attractiveness may indicate that engagement with digital environments is linked to the experience of being perceived and accepted within a relationship in ways that differ from the evaluation of one's own body. This may reflect more indirect mechanisms related to self-presentation, social comparison, or the perceived importance of attractiveness in social interactions (4). At the same time, the absence of similarly clear associations with other SAAR dimensions suggests that the role of media in shaping relational attractiveness remains limited and heterogeneous.

The role of aesthetic medicine procedures and professional activity in selected SAAR dimensions also deserves attention. Aesthetic procedures may reflect active investment in appearance and attempts to maintain a sense of attractiveness during a period marked by visible bodily changes associated with aging and hormonal transition. Previous studies suggest that such procedures may be associated with improvements in body image, body satisfaction, and self-esteem, and may also relate to women's social and relational functioning (24). At the same time, their use may co-occur with greater importance attributed to physical appearance in social and relational functioning and may be shaped by broader media and sociocultural pressures (7). Professional activity, in turn, may be interpreted as part of a broader context of social participation, autonomy, and well being in midlife women, although its links with appearance-related self-evaluation are likely to operate indirectly through psychosocial and health-related mechanisms (29, 30).

It is also important to note that the models related to SAAR explained only a small proportion of the variance. This suggests that self-perceived attractiveness in a partner relationship is a complex construct that likely depends more strongly on other factors, such as relationship quality, partner communication, partner support, psychological well being, and the severity of menopausal symptoms. From a public health perspective, this indicates that interpreting relational attractiveness solely through the lens of media-related variables would be insufficient. Rather, the present findings support the need to view women's functioning during the menopausal transition within a broader relational and psychosexual framework that integrates somatic, psychological, and social aspects of health.

### 4.3 Public health implications

The findings may have practical relevance for educational and psychoprophylactic interventions targeted at women in the menopausal transition. Support in this area should address not only exposure to digital media, but also the ways in which women interpret media messages related to appearance, aging, and femininity. The results also suggest that discussions of body image in the care of midlife women should extend beyond aesthetic concerns to include sexuality, relationship-related experiences, and the broader psychosocial context of menopausal changes. To mitigate appearance-related pressure and strengthen body-acceptance education in midlife women, brief media/digital literacy screening could be integrated into counseling and psychological support services. In practice, such screening could assess susceptibility to appearance-based social comparison, perceived impact of media portrayals, and engagement with appearance-focused content, enabling more tailored psychoeducation and preventive interventions.

### 4.4 Strengths and limitations

This study focused on women in the menopausal transition, a group still relatively underrepresented in research on body image and media-related influences. Body image was assessed multidimensionally, and the study distinguished between internet and social media use and the perceived influence of media portrayals of mature women. Another strength was the inclusion of aesthetic medicine procedures as a variable reflecting the contemporary sociocultural context of body image in midlife.

The study has several limitations. Its cross-sectional design does not allow causal inferences. Menopausal status was determined using interview-based STRAW+10 classification without hormonal confirmation, which may introduce misclassification. Media-related predictors were operationalized broadly: internet and social media use was measured with a dichotomous yes/no item and did not capture heterogeneity in frequency, duration, platform type, or appearance-focused engagement. This may have attenuated associations. In addition, we did not assess the type of content consumed; cognitive and affective responses may differ substantially depending on whether exposure involves educational or body-positive content vs. appearance-focused comparison content. Likewise, perceived media influence was self-reported and captured explicit perceptions rather than implicit processes such as appearance-based social comparison and internalization of appearance ideals. Use of aesthetic medicine procedures was also assessed broadly and did not capture frequency, timing, number of procedures, specific procedure types, or levels of invasiveness. The models, particularly for SAAR, explained only a small proportion of the variance, suggesting that additional relational and psychosexual factors not captured in the present study are likely important. Finally, because the study was conducted in one region of Poland and did not collect information on ethnicity or migration background, generalizability to more culturally diverse settings may be limited. Future studies should incorporate validated measures of digital/media literacy, content-specific engagement, and key mechanisms such as social comparison and internalization, as well as more comprehensive relational and psychosexual variables.

## 5 Conclusions

Among women in the menopausal transition, body image was associated with both media-related and non-media factors. Perceived media influence on body perception emerged as a more consistent correlate of BES dimensions than internet and social media use itself: it was negatively associated with Weight Concern and Physical Condition, whereas internet and social media use was significantly related only to Sexual Attractiveness. BMI, socioeconomic conditions, use of aesthetic medicine procedures, and age also remained significant in the final BES models. Media-related factors showed selective associations with self-attractiveness in a relationship: perceived media influence on body perception was associated with Body Acceptance and Appearance Evaluation, whereas internet and social media use was associated with Partner Acceptance. These findings support the need to distinguish between media exposure and subjective media impact in research and care focused on body image in midlife women. Given the cross-sectional design and broad media measures, these findings should be interpreted as associations and warrant replication using more granular, content-specific indicators of digital media exposure.

## Data Availability

The raw data supporting the conclusions of this article will be made available by the authors, without undue reservation.
