# Supplementary material for: Body image and self-attractiveness in a relationship during the menopausal transition: associations with digital media and perceived media influence
Source: Front Public Health. 2026 Jun 17;14:1829865. doi: 10.3389/fpubh.2026.1829865 (PMC13319057; doi:10.3389/fpubh.2026.1829865)
Supplement: Supplementary file 1 [file Table_1.docx]

**Supplementary Material 1. Full hierarchical regression models**

**Supplementary Table S1.** Full hierarchical linear regression models predicting the BES Sexual Attractiveness subscale.

| **Model** | | **B** | **SE** | **β** | **t** | **p** | **95%**  **CI LL** | **95%**  **CI UL** | **VIF** |
| --- | --- | --- | --- | --- | --- | --- | --- | --- | --- |
| **1** | (Constant) | 39.379 | 0.998 |  | 39.452 | 0.000 | 37.419 | 41.339 |  |
|  | Internet and social media use | 4.829 | 1.078 | 0.183 | 4.478 | **0.000** | 2.711 | 6.947 | 1.032 |
|  | Perceived media influence on body perception | -0.005 | 0.743 | 0.000 | -0.006 | 0.995 | -1.465 | 1.455 | 1.032 |
| F(2, 600) = 10.346, p < 0.001, R = 0.183, R² = 3.3%, adjusted R² = 3.0% | | | | | | | | | |
| **2** | (Constant) | 38.240 | 1.012 |  | 37.769 | 0.000 | 36.252 | 40.228 |  |
|  | Internet and social media use | 4.211 | 1.069 | 0.159 | 3.939 | **0.000** | 2.112 | 6.311 | 1.049 |
|  | Perceived media influence on body perception | 0.173 | 0.732 | 0.010 | 0.237 | 0.813 | -1.265 | 1.611 | 1.035 |
|  | BMI | 3.180 | 0.691 | 0.183 | 4.602 | **0.000** | 1.823 | 4.537 | 1.017 |
| F(3, 599) = 14.190, p < 0.001, R = 0.258, R² = 6.6%, adjusted R² = 6.2% | | | | | | | | | |
| **3** | (Constant) | 37.950 | 1.004 |  | 37.815 | 0.000 | 35.979 | 39.921 |  |
|  | Internet and social media use | 3.232 | 1.086 | 0.122 | 2.975 | **0.003** | 1.099 | 5.366 | 1.108 |
|  | Perceived media influence on body perception | -0.110 | 0.727 | -0.006 | -0.151 | 0.880 | -1.538 | 1.319 | 1.046 |
|  | BMI | 3.050 | 0.684 | 0.176 | 4.461 | **0.000** | 1.707 | 4.393 | 1.019 |
|  | Education | 2.740 | 0.706 | 0.158 | 3.884 | **0.000** | 1.355 | 4.126 | 1.084 |
| F(4, 598) = 14.664, p < 0.001, R = 0.299, R² = 8.9%, adjusted R² = 8.3% | | | | | | | | | |
| **4** | (Constant) | 37.957 | 0.997 |  | 38.088 | 0.000 | 36.000 | 39.914 |  |
|  | Internet and social media use | 3.127 | 1.079 | 0.118 | 2.897 | **0.004** | 1.007 | 5.247 | 1.110 |
|  | Perceived media influence on body perception | -0.583 | 0.739 | -0.032 | -0.789 | 0.430 | -2.034 | 0.868 | 1.093 |
|  | BMI | 2.824 | 0.683 | 0.163 | 4.134 | **0.000** | 1.482 | 4.165 | 1.031 |
|  | Education | 2.525 | 0.704 | 0.145 | 3.586 | **0.000** | 1.142 | 3.908 | 1.095 |
|  | Use of aesthetic medicine procedures | 2.936 | 0.956 | 0.124 | 3.070 | **0.002** | 1.058 | 4.814 | 1.083 |
| F(5, 597) = 13.781, p < 0.001, R = 0.322, R² = 10.3%, adjusted R² = 9.6% | | | | | | | | | |
| **5** | (Constant) | 36.797 | 1.060 |  | 34.728 | 0.000 | 34.716 | 38.878 |  |
|  | Internet and social media use | 2.682 | 1.082 | 0.101 | 2.479 | **0.013** | 0.557 | 4.806 | 1.130 |
|  | Perceived media influence on body perception | -0.570 | 0.734 | -0.031 | -0.777 | 0.437 | -2.011 | 0.871 | 1.093 |
|  | BMI | 2.564 | 0.683 | 0.148 | 3.752 | **0.000** | 1.222 | 3.907 | 1.047 |
|  | Education | 2.171 | 0.709 | 0.125 | 3.064 | **0.002** | 0.779 | 3.563 | 1.125 |
|  | Use of aesthetic medicine procedures | 3.210 | 0.954 | 0.135 | 3.365 | **0.001** | 1.336 | 5.083 | 1.093 |
|  | Self-rated socioeconomic conditions | 2.435 | 0.794 | 0.123 | 3.066 | **0.002** | 0.875 | 3.994 | 1.085 |
| F(6, 596) = 13.213, p < 0.001, R = 0.343, R²= 11.7%, adjusted R²=10.9% | | | | | | | | | |
| **6** | (Constant) | 47.727 | 5.328 |  | 8.958 | 0.000 | 37.264 | 58.191 |  |
|  | Internet and social media use | 2.427 | 1.085 | 0.092 | 2.236 | **0.026** | 0.295 | 4.559 | 1.144 |
|  | Perceived media influence on body perception | -0.580 | 0.731 | -0.032 | -0.792 | 0.429 | -2.016 | 0.857 | 1.093 |
|  | BMI | 2.384 | 0.687 | 0.137 | 3.470 | **0.001** | 1.035 | 3.733 | 1.064 |
|  | Education | 2.057 | 0.709 | 0.118 | 2.903 | **0.004** | 0.665 | 3.449 | 1.131 |
|  | Use of aesthetic medicine procedures | 3.005 | 0.956 | 0.127 | 3.143 | **0.002** | 1.127 | 4.883 | 1.104 |
|  | Self-rated socioeconomic conditions | 2.510 | 0.793 | 0.127 | 3.166 | **0.002** | 0.953 | 4.067 | 1.087 |
|  | Age | -0.209 | 0.100 | -0.083 | -2.093 | **0.037** | -0.406 | -0.013 | 1.066 |
| F(7, 595) = 12.015, p < 0.001, R = 0.352, R² = 12.4%, adjusted R² = 11.4% | | | | | | | | | |

*B – unstandardized coefficient; SE – standard error; β – standardized coefficient; 95% CI – 95% confidence interval; VIF – variance inflation factor; F – analysis of variance (ANOVA) statistic; R – multiple correlation coefficient; R² – coefficient of determination*

**Supplementary Table S2.** Full hierarchical linear regression models predicting the BES Weight Concern subscale.

| **Model** | | **B** | **SE** | **β** | **t** | **p** | **95%**  **CI LL** | **95%**  **CI UL** | **VIF** |
| --- | --- | --- | --- | --- | --- | --- | --- | --- | --- |
| **1** | (Constant) | 29.383 | 0.950 |  | 30.929 | 0.000 | 27.517 | 31.249 |  |
|  | Internet and social media use | 3.040 | 1.026 | 0.122 | 2.962 | **0.003** | 1.024 | 5.055 | 1.032 |
|  | Perceived media influence on body perception | -1.593 | 0.708 | -0.092 | -2.252 | **0.025** | -2.983 | -0.204 | 1.032 |
| F(2, 600) = 5.928, p = 0.003, R = 0.139, R² = 1.9%, adjusted R² = 1.6% | | | | | | | | | |
| **2** | (Constant) | 27.220 | 0.911 |  | 29.864 | 0.000 | 25.430 | 29.010 |  |
|  | Internet and social media use | 1.867 | 0.962 | 0.075 | 1.940 | 0.053 | -0.023 | 3.757 | 1.049 |
|  | Perceived media influence on body perception | -1.256 | 0.659 | -0.073 | -1.905 | 0.057 | -2.550 | 0.039 | 1.035 |
|  | BMI | 6.038 | 0.622 | 0.368 | 9.707 | **0.000** | 4.816 | 7.260 | 1.017 |
| F(3, 599) = 35.978, p < 0.001, R = 0.391, R² = 15.3%, adjusted R² = 14.8% | | | | | | | | | |
| **3** | (Constant) | 27.206 | 0.904 |  | 30.082 | 0.000 | 25.430 | 28.982 |  |
|  | Internet and social media use | 1.694 | 0.956 | 0.068 | 1.771 | 0.077 | -0.185 | 3.572 | 1.052 |
|  | Perceived media influence on body perception | -1.727 | 0.670 | -0.100 | -2.577 | **0.010** | -3.044 | -0.411 | 1.087 |
|  | BMI | 5.813 | 0.621 | 0.354 | 9.359 | **0.000** | 4.593 | 7.033 | 1.030 |
|  | Use of aesthetic medicine procedures | 2.795 | 0.866 | 0.125 | 3.227 | **0.001** | 1.094 | 4.495 | 1.073 |
| F(4, 598) = 30.010, p < 0.001, R = 0.409, R² = 16.7%, adjusted R² = 16.2% | | | | | | | | | |
| **4** | (Constant) | 26.248 | 0.973 |  | 26.966 | 0.000 | 24.337 | 28.160 |  |
|  | Internet and social media use | 1.133 | 0.976 | 0.045 | 1.160 | 0.247 | -0.785 | 3.050 | 1.107 |
|  | Perceived media influence on body perception | -1.669 | 0.667 | -0.097 | -2.501 | **0.013** | -2.980 | -0.358 | 1.088 |
|  | BMI | 5.673 | 0.621 | 0.346 | 9.143 | **0.000** | 4.455 | 6.892 | 1.038 |
|  | Use of aesthetic medicine procedures | 2.625 | 0.864 | 0.117 | 3.037 | **0.002** | 0.928 | 4.323 | 1.079 |
|  | Place of residence | 1.964 | 0.760 | 0.100 | 2.585 | **0.010** | 0.472 | 3.457 | 1.076 |
| F(5, 597) = 25.573, p < 0.001, R = 0.420, R² = 17.6%, adjusted R² = 16.9% | | | | | | | | | |
| **5** | (Constant) | 25.431 | 1.026 |  | 24.784 | 0.000 | 23.416 | 27.446 |  |
|  | Internet and social media use | 0.748 | 0.985 | 0.030 | 0.760 | 0.448 | -1.187 | 2.683 | 1.136 |
|  | Perceived media influence on body perception | -1.682 | 0.665 | -0.098 | -2.531 | **0.012** | -2.988 | -0.377 | 1.088 |
|  | BMI | 5.485 | 0.623 | 0.334 | 8.806 | **0.000** | 4.261 | 6.708 | 1.054 |
|  | Use of aesthetic medicine procedures | 2.792 | 0.864 | 0.125 | 3.233 | **0.001** | 1.096 | 4.488 | 1.086 |
|  | Place of residence | 1.894 | 0.757 | 0.096 | 2.501 | **0.013** | 0.407 | 3.382 | 1.078 |
|  | Self-rated socioeconomic conditions | 1.731 | 0.712 | 0.092 | 2.430 | **0.015** | 0.332 | 3.129 | 1.058 |
| F(6, 596) = 22.470, p < 0.001, R = 0.429, R² = 18.4%, adjusted R² = 17.6% | | | | | | | | | |

*B – unstandardized coefficient; SE – standard error; β – standardized coefficient; 95% CI – 95% confidence interval; VIF – variance inflation factor; F – analysis of variance (ANOVA) statistic; R – multiple correlation coefficient; R² – coefficient of determination*

**Supplementary Table S3.** Full hierarchical linear regression models predicting the BES Physical Condition subscale.

| **Model** | | **B** | **SE** | **β** | **t** | **p** | **95%**  **CI LL** | **95%**  **CI UL** | **VIF** |
| --- | --- | --- | --- | --- | --- | --- | --- | --- | --- |
| **1** | (Constant) | 27.832 | 0.786 |  | 35.393 | 0.000 | 26.288 | 29.376 |  |
|  | Internet and social media use | 2.532 | 0.850 | 0.123 | 2.981 | **0.003** | 0.864 | 4.201 | 1.032 |
|  | Perceived media influence on body perception | -1.064 | 0.586 | -0.075 | -1.817 | 0.070 | -2.214 | 0.086 | 1.032 |
| F(2, 600) = 5.301, p = 0.005, R = 0.132, R² = 1.7%, adjusted R² = 1.4% | | | | | | | | | |
| **2** | (Constant) | 26.680 | 0.788 |  | 33.838 | 0.000 | 25.132 | 28.229 |  |
|  | Internet and social media use | 1.908 | 0.833 | 0.092 | 2.292 | **0.022** | 0.273 | 3.543 | 1.049 |
|  | Perceived media influence on body perception | -0.884 | 0.570 | -0.062 | -1.550 | 0.122 | -2.004 | 0.236 | 1.035 |
|  | BMI | 3.215 | 0.538 | 0.237 | 5.976 | **0.000** | 2.159 | 4.272 | 1.017 |
| F(3, 599) = 15.642, p < 0.001, R = 0.270, R² = 7.3%, adjusted R² = 6.8% | | | | | | | | | |
| **3** | (Constant) | 26.665 | 0.779 |  | 34.246 | 0.000 | 25.136 | 28.194 |  |
|  | Internet and social media use | 1.721 | 0.823 | 0.083 | 2.090 | **0.037** | 0.104 | 3.338 | 1.052 |
|  | Perceived media influence on body perception | -1.392 | 0.577 | -0.098 | -2.412 | **0.016** | -2.525 | -0.259 | 1.087 |
|  | BMI | 2.973 | 0.535 | 0.219 | 5.560 | **0.000** | 1.923 | 4.023 | 1.030 |
|  | Use of aesthetic medicine procedures | 3.009 | 0.746 | 0.162 | 4.035 | **0.000** | 1.544 | 4.473 | 1.073 |
| F(4, 598) = 16.101, p < 0.001, R = 0.312, R² = 9.7%, adjusted R² = 9.1% | | | | | | | | | |
| **4** | (Constant) | 25.734 | 0.830 |  | 31.003 | 0.000 | 24.104 | 27.364 |  |
|  | Internet and social media use | 1.279 | 0.830 | 0.062 | 1.541 | 0.124 | -0.351 | 2.909 | 1.084 |
|  | Perceived media influence on body perception | -1.404 | 0.573 | -0.099 | -2.450 | **0.015** | -2.529 | -0.279 | 1.087 |
|  | BMI | 2.761 | 0.535 | 0.204 | 5.158 | **0.000** | 1.710 | 3.813 | 1.047 |
|  | Use of aesthetic medicine procedures | 3.184 | 0.743 | 0.172 | 4.288 | **0.000** | 1.726 | 4.642 | 1.079 |
|  | Self-rated socioeconomic conditions | 1.893 | 0.614 | 0.122 | 3.083 | **0.002** | 0.687 | 3.098 | 1.056 |
| F(5, 597) = 15.605, p < 0.001, R = 0.340, R² = 11.6%, adjusted R² = 10.8% | | | | | | | | | |
| **5** | (Constant) | 37.790 | 4.146 |  | 9.115 | 0.000 | 29.647 | 45.933 |  |
|  | Internet and social media use | 0.960 | 0.832 | 0.046 | 1.154 | 0.249 | -0.674 | 2.593 | 1.103 |
|  | Perceived media influence on body perception | -1.424 | 0.569 | -0.100 | -2.502 | **0.013** | -2.543 | -0.306 | 1.087 |
|  | BMI | 2.560 | 0.536 | 0.189 | 4.775 | **0.000** | 1.507 | 3.614 | 1.064 |
|  | Use of aesthetic medicine procedures | 2.939 | 0.742 | 0.159 | 3.959 | **0.000** | 1.481 | 4.397 | 1.092 |
|  | Self-rated socioeconomic conditions | 1.952 | 0.610 | 0.126 | 3.200 | **0.001** | 0.754 | 3.151 | 1.057 |
|  | Age | -0.231 | 0.078 | -0.117 | -2.967 | **0.003** | -0.384 | -0.078 | 1.060 |
| F(6, 596) = 14.377, p < 0.001, R = 0.356, R² = 12.6%, adjusted R² = 11.8% | | | | | | | | | |
| **6** | (Constant) | 37.018 | 4.140 |  | 8.942 | 0.000 | 28.888 | 45.149 |  |
|  | Internet and social media use | 0.514 | 0.847 | 0.025 | 0.607 | 0.544 | -1.150 | 2.178 | 1.154 |
|  | Perceived media influence on body perception | -1.376 | 0.567 | -0.097 | -2.427 | **0.016** | -2.490 | -0.263 | 1.088 |
|  | BMI | 2.453 | 0.536 | 0.181 | 4.579 | **0.000** | 1.401 | 3.505 | 1.071 |
|  | Use of aesthetic medicine procedures | 2.796 | 0.741 | 0.151 | 3.771 | **0.000** | 1.340 | 4.252 | 1.099 |
|  | Self-rated socioeconomic conditions | 1.895 | 0.608 | 0.122 | 3.117 | **0.002** | 0.701 | 3.089 | 1.059 |
|  | Age | -0.231 | 0.078 | -0.117 | -2.976 | **0.003** | -0.383 | -0.078 | 1.060 |
|  | Place of residence | 1.608 | 0.646 | 0.099 | 2.489 | **0.013** | 0.339 | 2.877 | 1.078 |
| F(7, 595) = 13.596, p < 0.001, R = 0.371, R² = 13.8%, adjusted R² = 12.8% | | | | | | | | | |
| **7** | (Constant) | 36.400 | 4.139 |  | 8.795 | 0.000 | 28.271 | 44.528 |  |
|  | Internet and social media use | 0.208 | 0.858 | 0.010 | 0.243 | 0.808 | -1.476 | 1.892 | 1.189 |
|  | Perceived media influence on body perception | -1.469 | 0.567 | -0.103 | -2.590 | **0.010** | -2.584 | -0.355 | 1.095 |
|  | BMI | 2.452 | 0.534 | 0.181 | 4.591 | **0.000** | 1.403 | 3.501 | 1.071 |
|  | Use of aesthetic medicine procedures | 2.643 | 0.743 | 0.143 | 3.558 | **0.000** | 1.184 | 4.102 | 1.110 |
|  | Self-rated socioeconomic conditions | 1.686 | 0.615 | 0.109 | 2.743 | **0.006** | 0.479 | 2.893 | 1.088 |
|  | Age | -0.218 | 0.078 | -0.111 | -2.815 | **0.005** | -0.371 | -0.066 | 1.066 |
|  | Place of residence | 1.505 | 0.646 | 0.092 | 2.330 | **0.020** | 0.236 | 2.775 | 1.084 |
|  | Education | 1.149 | 0.551 | 0.085 | 2.085 | **0.037** | 0.067 | 2.231 | 1.138 |
| F(8, 594) = 12.09, p < 0.001, R = 0.374, R² = 14.0%, adjusted R² = 12.8% | | | | | | | | | |

*B – unstandardized coefficient; SE – standard error; β – standardized coefficient; 95% CI – 95% confidence interval; VIF – variance inflation factor; F – analysis of variance (ANOVA) statistic; R – multiple correlation coefficient; R² – coefficient of determination*

**Supplementary Table S4.** Full hierarchical linear regression models predicting the SAAR Body Acceptance subscale.

| **Model** | | **B** | **SE** | **β** | **t** | **p** | **95%**  **CI LL** | **95%**  **CI UL** | **VIF** |
| --- | --- | --- | --- | --- | --- | --- | --- | --- | --- |
| **1** | (Constant) | 2.879 | 0.047 |  | 61.706 | 0.000 | 2.788 | 2.971 |  |
|  | Internet and social media use | 0.019 | 0.050 | 0.017 | 0.368 | 0.713 | -0.080 | 0.118 | 1.037 |
|  | Perceived media influence on body perception | 0.107 | 0.034 | 0.148 | 3.191 | **0.002** | 0.041 | 0.173 | 1.037 |
| F(2, 471) = 5.578, p = 0.004, R = 0.152, R² = 2.3%, adjusted R² = 1.9% | | | | | | | | | |
| **2** | (Constant) | 2.873 | 0.046 |  | 61.902 | 0.000 | 2.782 | 2.964 |  |
|  | Internet and social media use | 0.011 | 0.050 | 0.011 | 0.228 | 0.819 | -0.087 | 0.110 | 1.040 |
|  | Perceived media influence on body perception | 0.088 | 0.034 | 0.121 | 2.578 | **0.010** | 0.021 | 0.155 | 1.084 |
|  | Use of aesthetic medicine procedures | 0.118 | 0.043 | 0.126 | 2.717 | **0.007** | 0.033 | 0.203 | 1.054 |
| F(3, 470) = 6.230, p < 0.001, R = 0.196, R² = 3.8%, adjusted R² = 3.2% | | | | | | | | | |

*B – unstandardized coefficient; SE – standard error; β – standardized coefficient; 95% CI – 95% confidence interval; VIF – variance inflation factor; F – analysis of variance (ANOVA) statistic; R – multiple correlation coefficient; R² – coefficient of determination*

**Supplementary Table S5**. Full hierarchical linear regression models predicting the SAAR Appearance Evaluation subscale.

| **Model** | | **B** | **SE** | **β** | **t** | **p** | **95%**  **CI LL** | **95%**  **CI UL** | **VIF** |
| --- | --- | --- | --- | --- | --- | --- | --- | --- | --- |
| **1** | (Constant) | 3.063 | 0.051 |  | 59.757 | 0.000 | 2.962 | 3.164 |  |
|  | Internet and social media use | 0.021 | 0.055 | 0.018 | 0.382 | 0.703 | -0.088 | 0.130 | 1.037 |
|  | Perceived media influence on body perception | 0.090 | 0.037 | 0.114 | 2.443 | **0.015** | 0.018 | 0.163 | 1.037 |
| F(2, 471) = 3.352, p = 0.036, R = 0.118, R² = 1.4%, adjusted R² = 1.0% | | | | | | | | | |
| **2** | (Constant) | 2.981 | 0.063 |  | 47.636 | 0.000 | 2.858 | 3.104 |  |
|  | Internet and social media use | -0.007 | 0.056 | -0.006 | -0.118 | 0.906 | -0.118 | 0.104 | 1.089 |
|  | Perceived media influence on body perception | 0.090 | 0.037 | 0.114 | 2.453 | **0.015** | 0.018 | 0.163 | 1.037 |
|  | Employment status | 0.121 | 0.054 | 0.105 | 2.251 | **0.025** | 0.015 | 0.227 | 1.052 |
| F(3, 470) = 3.942, p = 0.009, R = 0.157, R² = 2.5%, adjusted R² = 1.8% | | | | | | | | | |

*B – unstandardized coefficient; SE – standard error; β – standardized coefficient; 95% CI – 95% confidence interval; VIF – variance inflation factor; F – analysis of variance (ANOVA) statistic; R – multiple correlation coefficient; R² – coefficient of determination*

**Supplementary Table S6.** Full hierarchical linear regression models predicting the SAAR Partner Acceptance subscale.

| **Model** | | **B** | **SE** | **β** | **t** | **p** | **95%**  **CI LL** | **95%**  **CI UL** | **VIF** |
| --- | --- | --- | --- | --- | --- | --- | --- | --- | --- |
| **1** | (Constant) | 2.930 | 0.054 |  | 54.661 | 0.000 | 2.825 | 3.035 |  |
|  | Internet and social media use | 0.161 | 0.058 | 0.129 | 2.777 | **0.006** | 0.047 | 0.274 | 1.037 |
|  | Perceived media influence on body perception | -0.026 | 0.039 | -0.032 | -0.682 | 0.495 | -0.102 | 0.050 | 1.037 |
| F(2, 471) = 3.869, p = 0.022, R = 0.127, R² = 1.6%, adjusted R² = 1.2% | | | | | | | | | |
| **2** | (Constant) | 2.326 | 0.278 |  | 8.355 | 0.000 | 1.779 | 2.873 |  |
|  | Internet and social media use | 0.177 | 0.058 | 0.142 | 3.047 | **0.002** | 0.063 | 0.291 | 1.054 |
|  | Perceived media influence on body perception | -0.022 | 0.039 | -0.026 | -0.566 | 0.571 | -0.098 | 0.054 | 1.040 |
|  | Age | 0.012 | 0.005 | 0.102 | 2.211 | **0.028** | 0.001 | 0.022 | 1.023 |
| F(3, 470) = 4.230, p = 0.006, R = 0.162, R² = 2.6%, adjusted R² = 2.0% | | | | | | | | | |
| **3** | (Constant) | 2.229 | 0.280 |  | 7.971 | 0.000 | 1.680 | 2.779 |  |
|  | Internet and social media use | 0.172 | 0.058 | 0.138 | 2.975 | **0.003** | 0.058 | 0.286 | 1.055 |
|  | Perceived media influence on body perception | -0.041 | 0.039 | -0.050 | -1.059 | 0.290 | -0.118 | 0.035 | 1.084 |
|  | Age | 0.013 | 0.005 | 0.117 | 2.532 | **0.012** | 0.003 | 0.024 | 1.041 |
|  | Use of aesthetic medicine procedures | 0.124 | 0.050 | 0.116 | 2.478 | **0.014** | 0.026 | 0.223 | 1.073 |
| F(4, 469) = 4.742, p < 0.001, R = 0.197, R² = 3.9%, adjusted R² = 3.1% | | | | | | | | | |

*B – unstandardized coefficient; SE – standard error; β – standardized coefficient; 95% CI – 95% confidence interval; VIF – variance inflation factor; F – analysis of variance (ANOVA) statistic; R – multiple correlation coefficient; R² – coefficient of determination*

**Supplementary Table S7.** Full hierarchical linear regression models predicting the SAAR Sexual Satisfaction subscale.

| **Model** | | **B** | **SE** | **β** | **t** | **p** | **95%**  **CI LL** | **95%**  **CI UL** | **VIF** |
| --- | --- | --- | --- | --- | --- | --- | --- | --- | --- |
| **1** | (Constant) | 3.022 | 0.057 |  | 53.353 | 0.000 | 2.911 | 3.134 |  |
|  | Internet and social media use | 0.012 | 0.061 | 0.010 | 0.203 | 0.839 | -0.108 | 0.133 | 1.037 |
|  | Perceived media influence on body perception | 0.002 | 0.041 | 0.003 | 0.056 | 0.955 | -0.078 | 0.083 | 1.037 |
| F(2, 471) = 0.025, p = 0.975, R = 0.010, R² = 0.0%, adjusted R² = -0.4% | | | | | | | | | |
| **2** | (Constant) | 3.152 | 0.069 |  | 45.463 | 0.000 | 3.016 | 3.288 |  |
|  | Internet and social media use | 0.005 | 0.061 | 0.004 | 0.079 | 0.937 | -0.114 | 0.124 | 1.038 |
|  | Perceived media influence on body perception | -0.008 | 0.041 | -0.009 | -0.186 | 0.852 | -0.087 | 0.072 | 1.043 |
|  | Having children | -0.150 | 0.047 | -0.146 | -3.180 | **0.002** | -0.243 | -0.057 | 1.009 |
| F(3, 470) = 3.388, p = 0.018, R = 0.145, R² = 2.1%, adjusted R² = 1.5% | | | | | | | | | |

*B – unstandardized coefficient; SE – standard error; β – standardized coefficient; 95% CI – 95% confidence interval; VIF – variance inflation factor; F – analysis of variance (ANOVA) statistic; R – multiple correlation coefficient; R² – coefficient of determination*
